# Size-controlled synthesis of monodispersed gold nanoparticles via carbon monoxide gas reduction

**DOI:** 10.1186/1556-276X-6-428

**Published:** 2011-06-16

**Authors:** Joseph K Young, Nastassja A Lewinski, Robert J Langsner, Laura C Kennedy, Arthi Satyanarayan, Vengadesan Nammalvar, Adam Y Lin, Rebekah A Drezek

**Affiliations:** 1 Department of Electrical and Computer Engineering, Rice University, MS-366, 6100 Main St., Houston, TX 77005, USA; 2 Department of Bioengineering, Rice University, MS-142, 6100 Main St., Houston, TX 77005, USA; 3 Department of Biochemistry and Cell Biology, Rice University, MS-140, 6100 Main St., Houston, TX 77005, USA

## Abstract

An in depth analysis of gold nanoparticle (AuNP) synthesis and size tuning, utilizing carbon monoxide (CO) gas as a reducing agent, is presented for the first time. The sizes of the AuNPs are tunable from ~4 to 100 nm by altering the concentration of HAuCl_4 _and inlet CO gas-injection flow rate. It is also found that speciation of aqueous HAuCl_4_, prior to reduction, influences the size, morphology, and properties of AuNPs when reduced with CO gas. Ensemble extinction spectra and TEM images provide clear evidence that CO reduction offers a high level of monodispersity with standard deviations as low as 3%. Upon synthesis, no excess reducing agent remains in solution eliminating the need for purification. The time necessary to synthesize AuNPs, using CO, is less than 2 min.

## Background

Metallic nanoparticles have attracted substantial attention due to their distinctive properties and various applications. AuNPs can exhibit a strong optical response to the extinction of surface plasmons by an alternating electromagnetic field [1]. By simply adjusting the size of the gold nanoparticles, this optical resonance can be positioned over hundreds of nanometers in wavelength across the visible into the near infrared spectrum [1,2]. Since these oscillations are located on the boundary of the metal and the external medium, these waves are very sensitive to changes in this boundary, such as the absorption of molecules to the metal surface [3]. These features render AuNPs useful as building blocks, and pave the way for fabricating biological labels, biological sensors, environmental detection of biological reagents, and clinical diagnostic technologies [4-6]. Many researchers have also exploited the unique optical properties of AuNPs for biomedical applications, such as thermal ablative cancer therapy and gene therapy [7-9].

Since the plasmon-derived optical resonance of gold nanoparticles is strongly related to the dimensions and morphology of the nanoparticles, the ability to synthesize monodispersed AuNPs is essential. The most popular and reliable method of producing AuNPs is an aqueous phase synthesis, which relies on the reduction of tetrachloroauric acid in the presence of a reducing agent to form colloid [10-15]. A number of different reducing agents can be used for the tetrachloroauric acid reduction. These agents have a significant influence on the morphology of the final product, and most of them lead to polydispersed nanoparticle solutions. To date, only a few methods have been established to synthesize AuNPs from about one nanometer to several hundred nanometers in diameter. A widely used method is based on the reduction of tetrachloroaurate ions in water using sodium citrate as a reductant to obtain AuNPs with diameters ranging from 16 to 147 nm [2,16,17]. While this method has demonstrated good quality control over particle size, a high level of monodispersity is limited to the synthesis of larger particles typically in the range of 22 to 120 nm. Another disadvantage to this synthesis method is that excess citrate remains in the solution. The residual citrate, which acts as a passivation layer on the surface of the nanoparticles, can reduce the effectiveness of surface functionalization with other biological markers [18].

Smaller-sized AuNPs, 1 to 5 nm, are usually prepared by borohydride reduction in the presence of thiol capping agents [19]. Disadvantages of this method include the use of toxic organic solvents and the potential presence of impurities introduced by using capping agents, which can also hinder the surface modification and functionality of particles for particular applications [20]. Also, AuNPs have been synthesized using formaldehyde as a reducing agent. One disadvantage is that formaldehyde is toxic, and the excess formaldehyde in the solution leads to solution instability and eventual particle aggregation [13].

Non-chemical based reduction methods, to synthesize AuNPs, have also been employed. Size-selected AuNPs have been synthesized by use of laser irradiation in a surfactant based aqueous environment [21]. Yet this method limits AuNPs sizes to sub 10 nm diameters. Meunier et al. were able to synthesize gold nanoparticles from 3 to ~80 nm via a femtosecond laser-assisted method [22]. An involved multi-step process, including a seeding step, was necessary to produce the larger particles. This process requires a complicated femtosecond laser setup and nanoparticle synthesis was also dependent on polymer utilization. Dispersed AuNPs were also synthesized using glow discharge plasma [23-26]. Researchers showed that this method can produce particles in less than 5 min yet these particles were limited to ~4 nm diameters [26]. Takai et al. used discharge plasma to produce larger AuNPs of irregular shapes [24]. Polydispersed spherical AuNPs, ~20 nm in diameter, were only produced after exposure times greater than 45 min.

As compared to the current synthesis methods, CO has an advantage in that no excess reducing agent remains in solution. This eliminates the need for purification via multiple centrifugation steps. The reduction of HAuCl_4 _with CO can also take place at room temperature, unlike other methods such as citrate reduction that require boiling of the solution. The time necessary to produce AuNPs using CO is less than 2 min compared to 20 min for comparable particle sizes using citrate reduction and 45 min for discharge plasma synthesis. CO reduction offers a cheap and flexible alternative to femtosecond laser-based AuNP synthesis processes while eliminating the need for surfactants and polymers to tune the nanoparticle sizes. To the best of our knowledge, there has never been an in depth study of AuNP synthesis, utilizing CO as a reducing agent, to enable size tuning from sub 5 to 100 nm diameters.

In this paper, an in depth analysis of AuNP synthesis utilizing CO gas as a reducing agent is presented. After synthesis, AuNP mono- and polydispersity was examined. The size and monodispersity of the AuNPs were tunable by controlling variables such as HAuCl_4 _concentration and gas flow during synthesis. The CO reduction method offered excellent tunability over a broad range of sizes while maintaining a high level of monodispersity. Ensemble extinction spectra and TEM images provide clear evidence that CO reduction offers excellent AuNP tunability and is a viable alternative to other synthesis methods.

## Results and discussion

AuNPs, synthesized by CO reduction, with average diameter ranging from 4 to 52 nm, were prepared as described below. A set of solutions consisting of HAuCl_4 _concentrations ranging from 0.01 mM up to 0.09 mM was used. Each HAuCl_4 _concentration was duplicated to ensure reproducibility. For each HAuCl_4 _concentration, five 40 mL samples were prepared. Each sample was aerated at different flow rates controlled by a control valve. The five solutions were exposed to CO gas at flow rates of 16.9, 25.45, 31.59, 37.0, and 42.9 mL/min, respectively. The effect of stirring speed was examined, and it was found that the number of revolutions per minute (rpm), by which the solution was stirred, played a role in particle size and morphology. The optimal stir speed, for producing the most monodispersed particles, was found to be 500 rpm. For the following discussion, each solution was constantly stirred at a rate of 500 rpm during synthesis unless noted otherwise. Additionally, the effect of gas-injection flow rates and diffuser pore size on nanoparticle monodispersity and reaction completion times were investigated. It was found that a 60-μm average diffuser pore size was sufficient for producing monodispersed particles. The solution temperature, prior to aeration, was maintained between 20 and 22°C.

### Formation of colloidal gold

The Au^3+ ^reduction, by CO, to Au^0 ^takes place via a number of redox reactions. When the CO gas is introduced into the aqueous HAuCl_4 _solution, electrons are donated to the [AuCl_4_]^- ^ions. For [AuCl_4_]^- ^ions to be reduced to gold atoms, a series of redox reactions take place. This includes the liberations of Cl^- ^ions and is described by Equations 1 and 2.(1)(2)

The electrons are contributed from the reaction of CO and dihydrogen monoxide and the reducing half reactions are given in Equations 3 and 4.(3)(4)

The thermodynamics of HAuCl_4 _reduction in aqueous solutions using CO is presented (see Additional file 1).

### Synthesis of AuNPs

**Figure 1:**
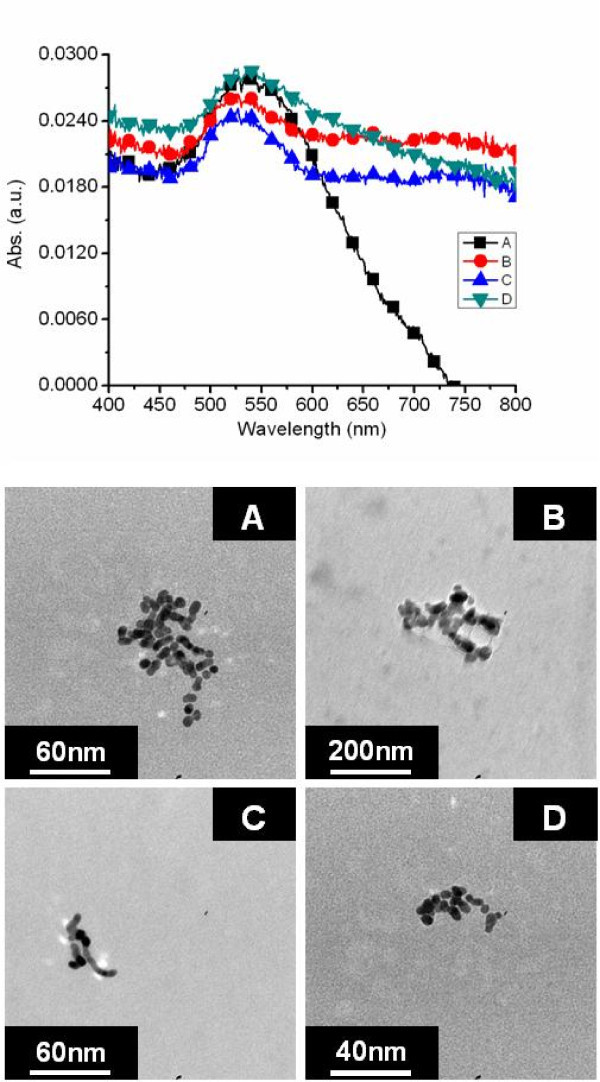
**Effect of CO flow rate on nanoparticle morphology**. UV-visible extinction spectra of nanoparticles synthesized from a chloroauric acid concentration of 0.01 mM aerated at flow rates of 16.9, 25.5, 37.0, and 42.9 mL/min corresponding to A, B, C, and D, respectively, with accompanying TEM micrographs. A smoother, more pronounced spectrum was generated at the minimum flow rate of 16.9 mL/min when compared to the other injection flow rates. As the flow rate was increased from 16.9 to 42.9 mL/min the change in spectral symmetry was clearly visible.

To illustrate the effects of CO gas flow injection rates on nanoparticle synthesis, nanoparticles were synthesized from an aqueous solution of HAuCl_4 _acid at a concentration of 0.01 mM. Even at this lower concentration, which is normally not used for the synthesis of AuNPs, the extinction spectra is clearly visible and well formed as evident in Figure 1. A smoother, more pronounced spectrum was generated at the minimum flow rate of 16.9 mL/min when compared to the other injection flow rates. As the flow rate was increased from 16.9 to 42.9 mL/min the change in spectral symmetry was clearly visible. TEM micrographs of the corresponding nanoparticles are displayed in Figure 1. The gas-injection flow rate of 16.9 mL/min produced individual nanoparticles compared to the other injection rates. The nanoparticles produced by the 16.9 mL/min flow rate ranged in size from 5 to 11 nm in diameter. A flow rate of 25.45 mL/min, Figure 1B, produced nanoparticle aggregates and irregularly shaped particulate matter. Nanoparticles synthesized at a flow rate of 31.59 mL/min consisted of aggregated particle chains. A CO flow rate of 37 mL/min (Figure 1C) resulted in aggregated particle chains similar to that of nanoparticles produced at a flow rate of 25.45 mL/min. The particle aggregation in Figure 1B, D was evident by the broad spectral band. As the flow rate increased to 42.9 mL/min, the nanoparticles became elliptical in shape and very polydispersed. The nanoparticle sizes, when aerated at 42.9 mL/min, ranged from 5 to 40 nm in diameter with some aggregated particles; this size distribution is reflected in the broad spectral band.

**Figure 2:**
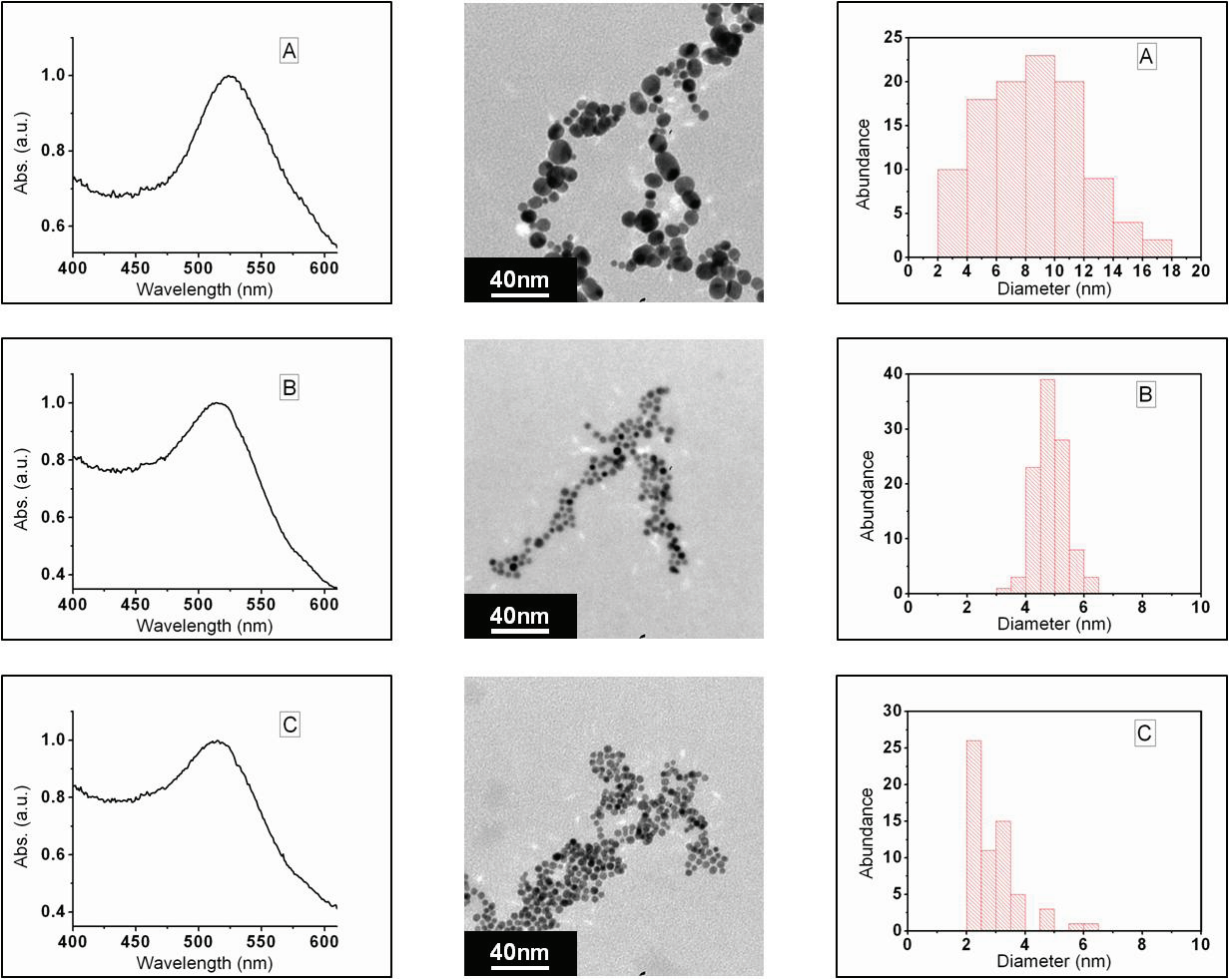
**Effect of CO flow rate on nanoparticle distribution**. UV-visible extinction spectra of nanoparticles synthesized from a chloroauric acid concentration of 0.03 mM aerated at flow rates of 16.9, 25.5, and 37.0 mL/min corresponding to A, B, and C, respectively, with accompanying TEM micrographs and histograms.

**Figure 3:**
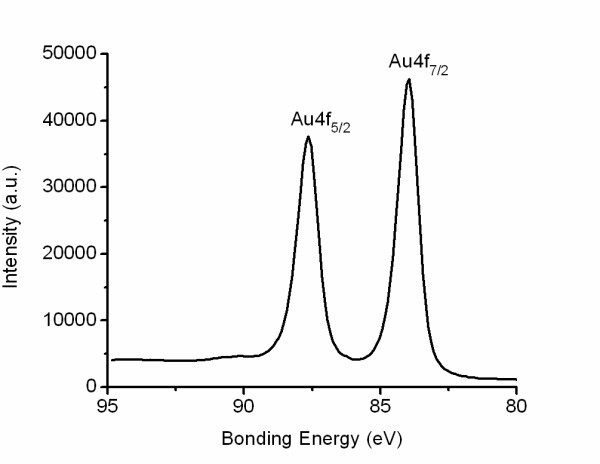
**XPS spectrum of AuNPs synthesized via CO gas reduction**. The Au 4f_7/2 _peak appeared at a binding energy of 83.98 eV and the Au 4f_5/2 _peak appeared at a binding energy of 87.71 eV. This indicates the formation of metallic gold [27,28].

Increasing the chloroauric acid concentration reduced the polydispersity of the nanoparticles, yet the gas-injection flow rate continued to influence the AuNP size distribution profiles. Figure 2 shows the UV-visible spectra of AuNPs synthesized from a chloroauric acid concentration of 0.03 mM at flow rates of 16.9, 25.5, and 37.0 mL/min (Figure 2A, B, C). The polydispersity of the AuNPs aerated at 16.9 mL/min (Figure 2A) is represented by a broad particle distribution curve. The particle sizes for Figure 2A ranged from 2.5 to 17 nm in diameter. Increasing the CO flow reduced the width of the particle distribution curve where an optimum inlet gas flow was obtained at 25.5 mL/min (Figure 2B). The standard deviation for 2B was 7%, well below the 13 to 15% normally obtained for comparable sizes via citrate reduction [3]. To confirm the formation of Au atoms from HAuCl_4_, the valence state of Au was identified by X-ray photoelectron spectroscopy (XPS). Figure 3 shows an XPS spectrum of AuNPs synthesized via CO gas reduction. The Au 4f_7/2 _peak appeared at a binding energy of 83.98 eV and the Au 4f_5/2 _peak appeared at a binding energy of 87.71 eV. This indicates the formation of metallic gold [27,28]. These particles remained stable in excess of 12 months when stored at 4°C.

A better understanding of the effect of the gas flow rates and chloroauric acid concentrations on nanoparticle synthesis can be obtained by considering the mechanisms involved in nanoparticle nucleation and growth. When aerating the aqueous HAuCl_4 _solution with CO gas, the precursor concentration increases continuously with increasing time. As the concentration reaches supersaturation, nucleation takes place and leads to a decrease in concentration. The continued decrease of the concentration is due to the growth of the particles. During the growth process, two growth mechanisms could take place or a combination of the two. The first growth mechanism is due to the formation of particles from coalescence of the nuclei only. The second growth mechanism is due to the coalescence of nuclei into simple and multiple twins with further growth from monomer attachment of Au atoms on the surface [16].

To produce monodispersed AuNPs with CO gas, the rate of nucleation must be high enough so that the precursor concentration does not continue to climb. Instead a large amount of nuclei are formed in a short period. Turkevich et al. found that the nucleation process consists of a polymerization step [29]. When the aqueous HAuCl_4 _solution is neutral or acidic, the nucleus is formed by gold organic polymer. While the aqueous HAuCl_4 _solution is alkaline, a polymerization of gold hydroxide takes place [16,30]. The rate of growth of these nuclei should be fast enough to decrease the concentration below the nucleation concentration rapidly. This results in the creation of a limited number of seed particles. The rate of growth must be slow enough that the growth period is long compared with the nucleation period. This produces AuNPs with narrowed size distributions which are the result of the limited nucleation period.

### Factors affecting AuNP synthesis

Since the morphology is found to depend strongly on injection flow rates and HAuCl_4 _concentrations, a relationship between the HAuCl_4 _concentration and gas-injection flow rates on particle monodispersity can be found. Solution stir speeds during synthesis were examined and it was found that stir speeds had an effect on synthesis and played a role in nanoparticle size disparities. Slow solution stir speeds had the biggest affect on solutions aerated at a flow rate of 16.9 mL/min or below. Increasing the stir speed of the solution aided in the solubility and dispersal of the CO gas molecules during synthesis. It was found that adjusting the gas-injection flow rate compensated for a reduction or increase in solution stir speed. The gas diffuser pore size affected the synthesis process considerably when the solution was at a standstill or stirred at a relatively slow speed below 75 rpm. Once the solution stir speed approached and/or crossed the 75 rpm threshold, injection-hole size produced only small variances. Once the stir speed reached 500 rpm, there was no difference between samples produced with different diffuser pore sizes, and only the Au concentration or gas-injection flow rates affected particle sizes. Therefore, the solution stirring speed was maintained at 500 rpm to isolate the gas-injection flow rate and Au concentration effect on nanoparticle synthesis.

A chloroauric concentration of 0.03 mM and an inlet gas flow of 16.9 mL/min stirred at 500 rpm resulted in coalescence and growth of nanoparticles before the nucleation reached equilibrium. In essence, the induction period was initiated with a slow autocatalytic rise in the number of nuclei due to the lack of sufficient reducing agent in the solution. Because of this slow nucleus formation, new nuclei were being formed while existing nuclei had already undergone coalescence resulting in polydispersity. Increasing the flow rate to 25.5 mL/min increased the autocatalytic rise in the number of nuclei. Particle growth took place after cessation of the nucleation process resulting in monodispersity. This is illustrated by the fact that the particle distribution curve for Figure 2B consisted of particle sizes in the range of 4 to 6 nm as opposed to the range of 2 to 17 nm (Figure 2A). By increasing the flow rate further (Figure 2C), rapid coalescence of the nuclei takes place. The resulting polydispersity of the sol at increased gas-injection flow rates is still marginal compared to the lower flow rate of 16.9 mL/min. When comparing the spectra of Figure 2A, B, C the more polydispersed sample possesses a broadened spectrum. This is illustrated in more detail (see Additional file 2).

### Increasing HAuCl_4 _concentration

When the chloroauric acid concentration approached 0.2 mM, the gas-injection flow rate had a less pronounced effect on the spectra symmetry yet the flow rate continued to dictate the monodispersity of the particles. When particles were synthesized from a chloroauric acid concentration of 0.3 mM, the most monodispersed sample was produced at a flow rate of 25.5 mL/min. The mean diameter for this sample was 9 nm with a standard deviation of 11%.

**Figure 4:**
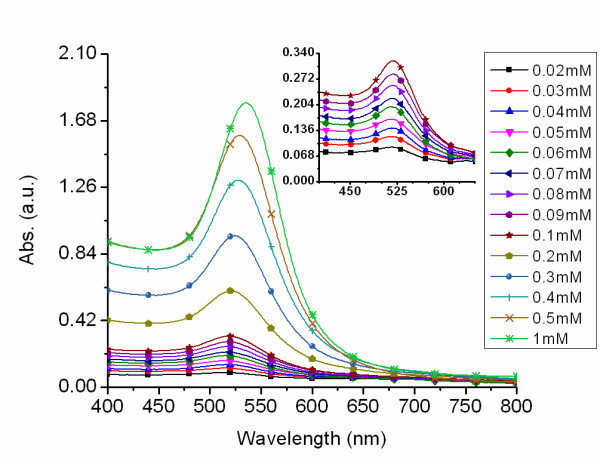
**Effect of increasing chloroauric acid concentrations on nanoparticle spectral profile**. UV-visible spectra of gold nanoparticles with increasing chloroauric acid concentrations from 0.02 to 0.05 mM in 0.01 mM increments, from 0.1 to 0.5 mM in .1 mM increments, and at 1 mM. The inset is the absorbance spectra of gold nanoparticles produced from concentrations of 0.02 to 0.1 mM.

**Figure 5:**
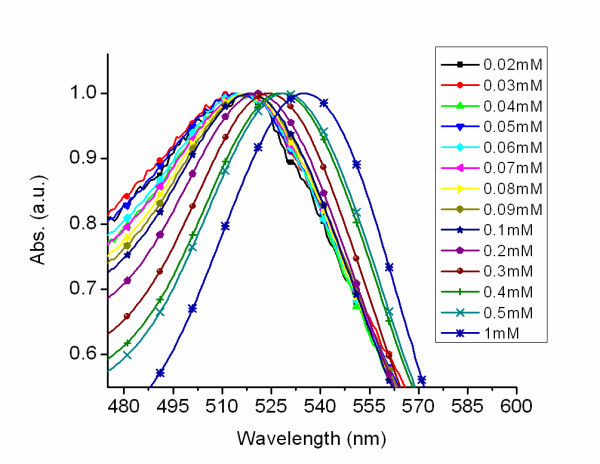
**Spectral shift based on chloroauric acid concentrations**. Normalized UV-visible spectra of gold nanoparticles with increasing chloroauric acid concentrations from 0.02 to 0.05 mM in 0.01 mM increments, from 0.1 to 0.5 mM in 0.1 mM increments, and at 1 mM. A red-shifting of the plasmon is observed as the chloroauric acid concentration is increased.

As the concentration increased to 0.5 mM, 20 to 25 nm particles were produced. Continual increase of the chloroauric acid concentration beyond 0.5 to 0.6 mM only produced small changes in nanoparticle size with increased absorbance. The standard deviation for the AuNPs produced at 0.6 mM was 8% indicating monodispersity. As the concentration was increased to 1 mM, nanoparticles approaching 30 nm in diameter were produced but the standard deviation approached 20%. Further doubling the concentration to 2 mM had no uniform effect on particle growth, with the majority of the particles in the 30 nm size regime and some of the particles in the 40 to 55 nm size regime with a standard deviation approaching 35%. The UV-visible spectra of the sol prepared at different concentrations (Figure 4), increasing from 0.02 to 1 mM, shows an increase in absorbance which correlates to an increase in particle concentration and volume. Figure 5 shows the pronounced red shifting of the plasmon, which is associated with increased nanoparticle size. The red shift of the plasmon is further illustrated (see Additional file 3). This shifting effect is in line with the prediction described by Mie theory [1,2]. The statistical analysis of the particles synthesized from aqueous solutions of HAuCl_4 _ranging from 0.02 to 0.6 mM revealed an average standard deviation of approximately 11%.

### Influence of pH on AuNP synthesis

It is known that pH is a factor influencing the nucleation and growth of AuNPs [13,16,30]. Since the synthesis process takes place in an acidic environment, the particle is formed from gold polymer with a small contribution from gold hydroxide polymer reduction. As the concentration of chloroauric acid increases, the pH of the solution decreases (see Additional file 4) resulting in particle formation solely by gold polymer reduction. In an acidic environment, the effective monodispersed particle size threshold was reached at approximately 25 nm. The effective monodispersed threshold was defined as a standard deviation below 13%. As previously mentioned, continual increase of the chloroauric concentration eventually resulted in adverse affects on nanoparticle monodispersity. To further grow particles and maintain monodispersity, HAuCl_4 _hydrolysis was explored. The addition of potassium carbonate (K_2_CO_3_) to generate an alkaline solution for gold hydroxide polymer reduction was systematically investigated. It was found that the speciation of HAuCl_4 _had great influence on the size and monodispersity of the AuNPs. As the pH increased, speciation of aqueous HAuCl_4 _occurred.

Adding K_2_CO_3 _raised the pH of the solution by allowing hydrolysis of HAuCl_4 _to take place to form gold hydroxide solution. A 200 mL aqueous HAuCl_4 _solution, with a concentration of 0.1 mM, was prepared by adding fresh gold to 200 mL of DI water. This solution was aged in an amber bottle, and light protected in a 4°C environment for a minimum of 72 h prior to use. A 0.5 N stock solution of K_2_CO_3 _was prepared and stirred for a minimum of 1 h. After aging, the chloroauric acid solution was allowed to gradually rise to 22°C. The pH was measured to be 3.6. HAuCl_4 _(0.1 mM) aqueous solution with various pH values were prepared by the addition of K_2_CO_3 _aqueous solution into 20 mL of HAuCl_4 _aqueous solution and shaken vigorously for a minimum of 1 min. This solution was allowed to age for 15 min before introduction of CO gas. The pH values of the aqueous solutions, measured prior to reduction, ranged from 4.25 to 11.4.

**Figure 6:**
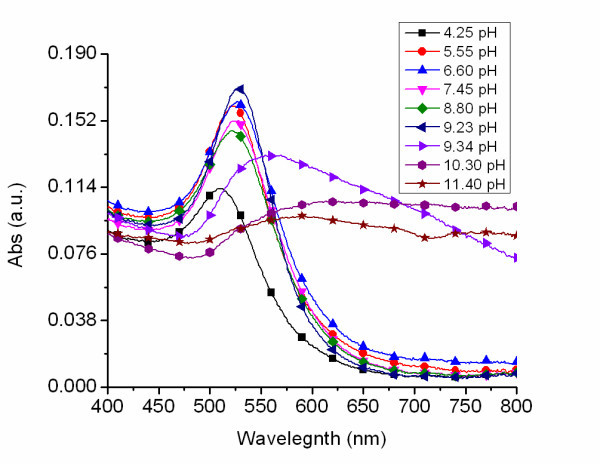
**Effect of pH on nanoparticle spectrum**. UV-visible spectra of AuNPs produced from a 0.1 mM HAuCl_4 _aqueous solution synthesized at varying pH values.

Figure 6 shows UV-visible absorption spectra of AuNPs prepared by reduction of hydrolyzed HAuCl_4 _at various pH. At pH = 4.25, the acquired AuNPs exhibited a symmetric spectrum with a surface plasmon resonance (SPR) peak at 512 nm. When the pH increased to 6.6, there was a SPR shift to 527 nm. When the pH increased to 7.45, the SPR peak position did not change much at 528 nm, and the SPR peak remained symmetric. The SPR feature changed abruptly when the pH was 9.34 showing a broad feature originating at 559 nm. The SPR peak red-shifted further when the pH increased to 10.3. Absorption in the NIR region also gained significant intensity.

Previous experimental and theoretical results demonstrated that AuCl_4 _undergoes a pH-dependant stepwise hydrolysis which gives way to [AuCl*_x_*(OH)_4-*x*_]^- ^[30,31]. The extent of hydrolysis in turn depends on the pH which gives an indication of the amount of OH^- ^available for hydrolysis. When the pH is low, [AuCl_4_]^- ^ions dominate the solution. As the pH is increased to 4.25, [AuCl_4_]^- ^concentration is lowered and the contribution from [AuCl_3_(OH)]^- ^ions is increased. Raising the pH of the solution to 6.66 reduced the concentration of [AuCl_4_]^- ^and [AuCl_3_(OH)]^- ^significantly, and the ionic composition was primarily made up of [AuCl_2_(OH)_2_]^- ^ions. Further increasing the pH to 8.8 resulted in large ion contribution from [AuCl(OH)_3_]^- ^ions. Additional increase to 10.3 resulted in an overwhelming ion contribution from [Au(OH)_4_]^- ^ions with an appreciable contribution from [AuCl(OH)_3_]^- ^ions. This was because [Au(OH)_4_]^- ^is amphoteric. Its solubility increased due to the formation of [Au(OH)_4_]^- ^at higher pH, thus making the soluble [Au(OH)_4_]^- ^the most dominant species at high pH instead of the precipitating [AuCl(OH)_3_]^- ^[30]. It is the control of hydrolysis to tune the speciation of [AuCl*_x_*(OH)_4-*x*_]^- ^that subsequently influenced the nanoparticle size.

It was observed that amongst the six species of [AuCl*_x_*(OH)_4-*x*_]^- ^discussed earlier, [Au(OH)_4_]^- ^seems to have the lower tendency to be reduced in solution to form colloidal gold. This was evident from its slow and gradual color change when reduced, taking approximately 7 min for complete reduction to occur. This was in contrast to the reduction of other [AuCl*_x_*(OH)_4-*x*_]^- ^species formed at lower pH where it was observed that the addition of CO gas caused a color change within seconds and total reduction within approximately 2 min. This observation may possibly be attributed to a weaker reduction potential of [Au(OH)_4_]^- ^compared to other species. It was found that adjustment to pH < 10 by addition of smaller amounts of K_2_CO_3 _resulted in the formation of other dominant species that had greater tendency to be reduced in solution to form colloidal gold. It was observed that the synthesis environment also affected nanoparticle stability. The stability of the nanoparticles was monitored for approximately 2 months to examine the pH effect on nanoparticle stability. As the pH increased, prior to synthesis, the nanoparticles became less stable. Table 1 illustrates the stability of the AuNP solutions produced at varying pH.

**Table 1:** Influence of pH upon stability of AuNPs

pH Before synthesis	pH After synthesis	Color	Stability After 1 h stored at 22°C	Stability After 6 h stored at 22°C	Stability After 2 months Stored at 4°C
4.25	3.72	Light pink	Stable	Stable	Small aggregation
4.25	3.72	Light pink	Stable	Stable	Small aggregation
5.55	4.75	Light red	Stable	Stable	Small aggregation
6.6	5.92	Light red	Stable	Stable	Stable
7.45	6.11	Light red	Stable	Stable	Stable
8.8	6.42	Light red	Stable	Stable	Stable
9.23	6.55	Medium red	Stable	Stable	Stable
9.34	6.32	Purple	Stable	Stable	Medium aggregation
10.3	8.10	Blue	Stable	Some aggregation	Heavy aggregation
11.4	10.96	Light blue	Crashed	Crashed	Crashed

It was observed that hydrolysis of [AuCl_4_]^- ^started to occur within minutes after the addition of K_2_CO_3 _indicating immediate formation of the [AuCl*_x_*(OH)_4-*x*_]^- ^species. It was further observed that Au colloid, of varying sizes, were produced when K_2_CO_3 _and HAuCl_4 _concentrations and gas-injection flow rates remained constant and only aging times varied. This indicated that aging the gold hydroxide solution, before the addition of CO gas, had a strong influence on the outcome of the reaction.

**Figure 7:**
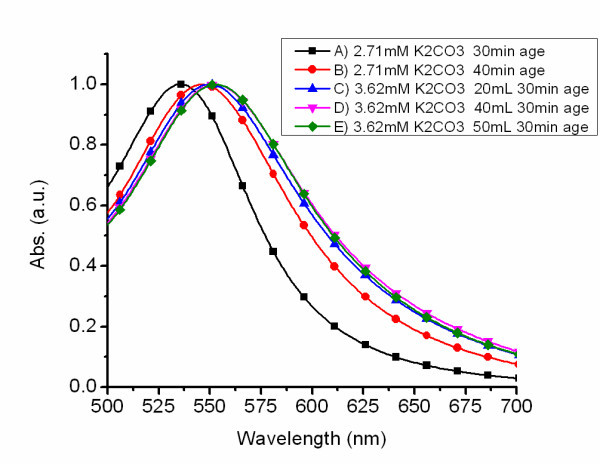
**Nanoparticle spectra as a function of K_2_CO_3 _concentration and aging**. UV-visible spectra of AuNPs produced from a mixture of 0.38 mM HAuCl_4 _aqueous solution with 2.71 mM or 3.62 mM K_2_CO_3_. A and B are 2.71 mM K_2_CO_3 _aged at 30 and 40 min, respectively, at an aeration volume of 40 mL. C, D, and E are 3.62 mM K_2_CO_3 _aged for 30 min each at aeration volumes of 20, 40, and 50 mL, respectively. All samples were aerated at a gas flow rate of 25.5 mL/min.

By controlling the development of the [AuCl*_x_*(OH)_4-*x*_]^- ^species, colloids of various sizes can be synthesized using CO as a reducing agent. When the pH is sufficiently high, the resultant aging process can generate coalescence of Au(OH)_4 _initiating a limited nucleation process absent of a reducing agent. This nucleation process is out of favor with the requirements necessary for generating monodispersed nanoparticles. Thus proper aging times must be determined to synthesize monodispersed nanoparticles of a particular size from a given K_2_CO_3 _and HAuCl_4 _concentration. Exploiting the control of [AuCl*_x_*(OH)_4-*x*_]^- ^species development, by addition of K_2_CO_3 _and aging of the solution, Au colloid in the ranges of 15 to 100 nm in diameter were produced. Spectra A and B in Figure 7 show the UV-visible spectra of Au colloid produced from a mixture of 200 mL 0.38 mM HAuCl_4 _aqueous solution and K_2_CO_3 _(2.71 mM) aged at 30 and 40 min, respectively, in solution reduction volumes of 40 mL. Both SPR peaks were well ordered with a SPR peak at 536 nm for the 30-min aged solution and 546 nm for the 40-min aged solution. Both solutions were aerated with CO gas at an inlet gas flow rate of 25.5 mL/min. The red-shift and dampening of the SPR peak indicated an increase in particle size. The effect of the solution volume being aerated was explored to determine if the amount of solution being aerated had an effect on nanoparticle size and monodispersity. Spectra C, D, and E in Figure 7 were produced from AuNPs synthesized from a 200 mL 0.38 mM HAuCl_4 _aqueous solution with K_2_CO_3 _(3.62 mM) aged for 30 min. The aeration volumes were 20, 40, and 50 mL, respectively. The amount of solution aerated had a small but noticeable effect on SPR peak position. The resulting SPR peak positions were 550, 553, and 554 nm for aeration volumes of 20, 40, and 50 mL, respectively. Increasing the amount of K_2_CO_3_, in a HAuCl_4 _aqueous solution of known concentration, while decreasing the aging time, produced larger AuNPs while still maintaining monodispersity. Aqueous solutions of 200 mL 0.38 mM HAuCl_4 _with 2.71 and 3.62 mM of K_2_CO_3 _aged for 30 min each produced AuNPs with SPR peak positions at 536 and 553 nm, respectively.

**Figure 8:**
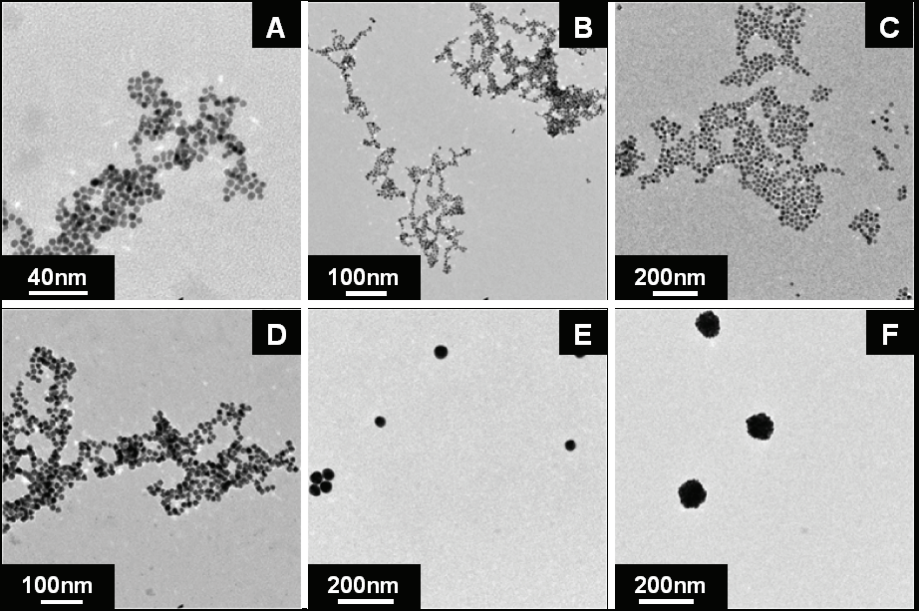
**TEM images of AuNPs synthesized by CO reduction of HAuCl_4_**. A, B, C, and D are TEM images of AuNPs synthesized without the addition of K_2_CO_3_. E and F are AuNPs synthesized from a hydrolyzed solution of aqueous HAuCl_4 _via the addition of K_2_CO_3_. The corresponding sizes of the AuNPs are 4, 6, 15, 25, 50, and ~100 nm respectively.

By employing a combination of gold polymer reduction and gold hydrolyzed polymer reduction, particles sizes from ~4 to 100 nm can be synthesized. Figure 8 shows a TEM micrograph illustrating the different sizes available using CO as a reducing agent. Figures 8A, B, C, D are TEM images of AuNPs synthesized without the addition of K_2_CO_3_. Figures 8E, F are AuNPs synthesized from a hydrolyzed solution of aqueous HAuCl_4 _via the addition of K_2_CO_3_. The corresponding sizes of the AuNPs are 4, 6, 15, 25, 50, and ~100 nm with standard deviations of 7, 13, 8, 8, 10, and 11%, respectively.

## Conclusions

These results indicate that AuNPs can be synthesized using CO as a reducing agent. CO offers tunability of nanoparticle sizes via altering HAuCl_4 _concentration and flow rate. The fast synthesis rates, ease of tunability, and absence of cytotoxic by products allow for these CO-based AuNPs to be optimized and readily produced for biomedical and industrial applications. The manipulation of the solution pH and speciation of HAuCl_4 _to control particle morphology may also be used as a means to tune the particle size. TEM micrographs and UV-visible spectral analysis confirm that the CO-based AuNPs are monodispersed upon synthesis. Future work will focus on how temperatures, upon synthesis, affect morphology. Nanoparticle surface chemistry and functionalization will also be explored. Cytotoxicity of the CO-based AuNPs in human cell lines will subsequently be investigated and compared against citrate-based nanoparticles.

## Methods

### Chemicals and materials

Hydrogen tetrachloroaurate III trihydrate (HAuCl_4_·3H_2_O, 99.99%), and absolute ethanol (C_2_H_5_OH, 99.5%) where purchased from Sigma Aldrich and used as received. Carbon monoxide (CO, 99%) was supplied by Matheson-Trigas. All solutions were prepared using ultrapure water (18 Mohm Millipore Milli-Q water).

### Pre-synthesis

All chloroauric acid solutions were aged in individual amber bottles under 4°C and light protected for a minimum of 3 days prior to use. All glassware used in the following procedures were cleaned in a bath of freshly prepared aqua regia solution (3 parts HCL acid to 1 part HNO_3 _acid) and rinsed thoroughly with ethanol three times and then rigorously rinsed four times with copious amounts of pure grade water and oven dried prior to use. Stirring was conducted by a PTFE-coated magnetic stir bar which was cleaned and dried in the same manner as the glassware.

### Carbon monoxide-based synthesis of pure aqueous HAuCl_4 _solution

Several chloroauric acid solutions were prepared for utilization with CO reduction. Various weights of fresh chloroauric acid were dissolved in individual amber bottles containing water (200 mL). At least two separate batches of all solution concentrations were employed to confirm reproducibility. One set of solutions consisted of varying concentrations of chloroauric acid (0.01 to 0.09 mM in 0.01 mM increments) and HAuCl_4 _(1 mM) and HAuCl_4 _(2 mM) solutions were prepared. A solution of HAuCl_4 _(1 wt%) was also prepared. Gold nanoparticles synthesized by CO reduction, with average diameter nanoparticles ranging from 4.5 to 52 nm were prepared as described below. For each HAuCl_4 _concentration five volumes (40 mL) were prepared. Each sample was aerated at different flow rates controlled by a control valve. The gas entered the solution via a 60 um pore gas diffuser (Fisher Scientific) attached to the end of the gas supply line downstream of the control valve. The five solutions were exposed to CO gas at flow rates of 16.9, 25.45, 31.59, 37.0, and 42.9 mL/min, respectively. The solution temperature, prior to aeration, was maintained between 20 and 22°C.

### Carbon monoxide-based synthesis of pH adjusted HAuCl_4 _solution and hydrolyzed HAuCl_4 _solution

A 200 mL aqueous HAuCl_4 _solution, with a concentration of 0.1 mM, was prepared by adding fresh gold to 200 mL of ultrapure Milli-Q water. This solution was aged in an amber bottle in light protected 4°C environment for a minimum of 72 h prior to use. After aging, the chloroauric acid solution was allowed to gradually rise to 22°C. A fresh stock solution of potassium carbonate (0.5 N) was prepared and stirred for a minimum of 1 h. HAuCl_4 _aqueous solutions with various pH values were prepared by the addition of certain amounts of K_2_CO_3 _aqueous solution into of HAuCl_4 _(0.1 mM) aqueous solution (20 mL) and shaken vigorously for a minimum of 1 min. This solution was allowed to age for 15 min before introduction of CO gas. The pH values of the aqueous solutions, measured prior to reduction, ranged from 4.25 to 11.4. Additionally several aqueous HAuCl_4 _(0.38 mM) solutions (200 mL) were prepared by adding fresh gold to ultrapure Milli-Q water (200 mL). These solutions were allowed to age for a minimum of 72 h. K_2_CO_3 _(75 mg, 2.71 mM) was added to two HAuCl_4 _(0.38 mM) solutions (200 mL) and aged for 30 and 40 min, respectively. K_2_CO_3 _(100 mg, 3.62 mM) was added to a HAuCl_4 _(0.38 mM) solution (200 mL) and aged for 30 min. All solutions were aerated with CO gas at an inlet flow rate of 25.5 mL/min.

### Nanoparticle characterization

Sample size distributions were determined by transmission electron microscopy (TEM) performed using a JEOL 1230 High Contrast-Transmission Electron Microscope (HC-TEM) operating between 80 and 100 kV. Samples were prepared for both instruments by contacting a AuNP (10 μL) drop with a carbon film coated 200 mesh copper grid. The grids were placed in a spotlessly clean container, covered and allowed to dry completely before use.

The optical response of the gold nanoparticles was determined by examining the optical extinction spectra of aqueous samples in 1 cm path length polystyrene cuvettes using a Varian Cary 300 UV-visible spectrophotometer. The UV-visible spectra were acquired at wavelengths between 400 to 800 nm. Distilled water was used as the reference and the blank for baseline subtraction.

XPS was carried out using a PHI Quantera SXM system. The soft X-ray source consisted of aluminum with source energy of 1486.7 eV. The take off angle was set at 45°. Precut silicon wafers 4.5 mm × 5 mm were cleaned by immersion in a 3:1 H_2_SO_4_:H_2_O_2 _(piranha) solution for 15 min and rinsed with ultrapure Milli-Q water then dried. The sample was prepared by concentrating the AuNPs and dropping colloidal solution on precut silicon wafers. They were placed in a spotlessly clean container, covered and allowed to dry.

## Abbreviations

CO: carbon monoxide; AuNP: gold nanoparticle; HC-TEM: high contrast-transmission electron microscope; rpm: revolutions per minute; SPR: surface plasmon resonance; TEM: transmission electron microscopy; XPS: X-ray photoelectron spectroscopy.

## Competing interests

The authors declare that they have no competing interests.

## Authors' contributions

JKY is the primary author and conceived of the study, carried out the conception and design, synthesis and experiments, characterization, acquisition of data, analysis and interpretation of data, and drafting of the manuscript. NAL and RJL equally contributed as secondary authors by conducting cytotoxicity studies, data analysis and manuscript revisions. LCK carried out experiments, performed particle characterizations and aided in the drafting of the manuscript. AS participated in the design of the study, carried out synthesis and experiments and helped draft the manuscript. AYL and VN participated in the design of the study and coordination. RAD is the principal investigator.

## Supplementary Material

Additional file 1**Thermodynamics of HAuCl_4 _reduction in aqueous solutions using carbon monoxide as a reducing agent**. The entire process is performed between 20 and 22°C and a pressure of 1 atm. The pH of the solution varies as a function of HAuCl_4 _concentration. Nernst equation describes potential of electrochemical cell as a function of concentrations of ions taking part in the reaction:(1a)where *E*^0 ^is the standard reduction potential, *R *is the absolute gas constant = 8.31441 J/(mol K), *F *is Faraday constant = 96484.6 C/mol, *T *is the absolute temperature = 295.15 K, *n *is number or electrons, and *Q *is the reaction quotient. *RT*/*F *can be considered constant.(2a)(3a)**The CO gas is injected at a flow rate of 25**.45 mL/min in 40 mL aqueous sample volumes. A water saturation constant of 0.26 g per 1 kg at 22°C is used.(4a)(5a)(6a)(7a)(8a)(9a)**Redox potentials (7) and (8) are given at pH 0**. The redox potentials are pH-dependent and must be adjusted for the varying pH values.Click here for file

Additional file 2 **Effect of CO flow rate on nanoparticle spectral profile**. Normalized UV-visible spectra of nanoparticles synthesized from a chloroauric acid concentration of 0.03 mM aerated at flow rates of 16.9, 25.5, and 37.0 mL/min corresponding to A, B, and C, respectively. The effect of the gas flow rate during synthesis is illustrated by a comparison of the three spectra. Click here for file

Additional file 3 **Plasmon peak position and absorbance value as a function of chloroauric acid concentration**. The chloroauric acid concentration ranging from 0.01 to 1 mM. The data is plotted on a logarithmic scale. As the HAuCl_4 _concentration increases the absorbance intensity increases with an accompanying red-shift of the plasmon peak position. Click here for file

Additional file 4 **pH values before and after AuNP synthesis**. pH values for given HAuCl_4 _concentrations ranging from 0.02 to 0.1 mM in 0.01 mM increments and from 0.1 to 0.5 mM in 0.1 mM increments. The *x*-axis is plotted on a logarithmic scale. The inset shows the pH values of the AuNP solutions from 0.01 to 0.1 mM and is plotted on a linear scale. As the reduction of HAuCl_4 _by CO takes place, H^+ ^ions are liberated decreasing the pH of the solution. All pH measurements were taken at room temperature. Click here for file
