# Supplementary material for: Size-controlled synthesis of monodispersed gold nanoparticles via carbon monoxide gas reduction
Source: Nanoscale Res Lett. 2011 Jun 16;6(1):428. doi: 10.1186/1556-276X-6-428 (PMC3211845; doi:10.1186/1556-276X-6-428)
Supplement: Additional file 1 — Thermodynamics of HAuCl4 reduction in aqueous solutions using carbon monoxide as a reducing agent. The entire process is performed between 20 and 22°C and a pressure of 1 atm. The pH of the solution varies as a function of HAuCl4 concentration. Nernst equation describes potential of electrochemical cell as a function of concentrations of ions taking part in the reaction:(1a) where E0 is the standard reduction potential, R is the absolute gas constant = 8.31441 J/(mol K), F is Faraday constant = 96484.6 C/mol, T is the absolute temperature = 295.15 K, n is number or electrons, and Q is the reaction quotient. RT/F can be considered constant.(2a)(3a) The CO gas is injected at a flow rate of 25.45 mL/min in 40 mL aqueous sample volumes. A water saturation constant of 0.26 g per 1 kg at 22°C is used.(4a)(5a)(6a)(7a)(8a)(9a) Redox potentials (7) and (8) are given at pH 0. The redox potentials are pH-dependent and must be adjusted for the varying pH values. [file 1556-276X-6-428-S1.DOC]

**Thermodynamics of HAuCl_4_ reduction in aqueous solutions using carbon monoxide as a reducing agent.**

The entire process is performed between 20 and 22°C and a pressure of 1 atm. The pH of the solution varies as a function of HAuCl_4_ concentration. Nernst equation describes potential of electrochemical cell as a function of concentrations of ions taking part in the reaction:

$E=E^{0}-\frac{RT}{nF}\ln(Q)$ (1)

where *E*^0^ is the standard reduction potential, *R* is the absolute gas constant = 8.31441 J/(mol K), *F* is Faraday constant = 96484.6 C/mol, *T* is the absolute temperature = 295.15 K, *n* is number or electrons, and *Q* is the reaction quotient. *RT*/*F* can be considered constant.

$Q=\frac{\left\{C\right\}^{c}\left\{D\right\}^{d}}{\left\{A\right\}^{a}\left\{B\right\}^{b}}$ (2)

$E=E^{0}-\frac{RT}{nF}*2.303*\log(Q)$ (3)

The CO gas is injected at a flow rate of 25.45 mL/min in 40 mL aqueous sample volumes. A water saturation constant of 0.26 g per 1 kg at 22°C is used.

$\text{Au}^{3+}+2e^{-}\to\text{Au}^{0} E^{0}(V)=1.52$ (4)

$\text{AuCl}_{4}^{-}+2e^{-}\to\text{AuCl}_{2}^{-}+2\text{Cl}^{-} E^{0}(V)=0.926$ (5)

$\text{AuCl}_{2}^{-}+e^{-}\to\text{Au}^{0}+2\text{Cl}^{-} E^{0}(V)=1.154$ (6)

$\text{AuCl}_{4}^{-}+3e^{-}\to\text{Au}^{0}+4\text{Cl}^{-} E^{0}(V)=1.002$ (7)

$\text{CO}(g)+\text{H}_{2}\text{O}\to\text{CO}_{2}(\text{aq})+2e^{-}+2\text{H}^{\text{+}} E^{0}(V)=0.11$ (8)

$\text{CO}(g)+2\text{H}_{2}\text{O}\to\text{HCO}_{3}^{-}+2e^{-}+3\text{H}^{\text{+}} E^{0}(V)=-0.101$ (9)

Redox potentials (7) and (8) are given at pH 0. The redox potentials are pH-dependent and must be adjusted for the varying pH values.
